# Human Probiotic *Lactobacillus paracasei*-Derived Extracellular Vesicles Improve Tumor Necrosis Factor-α-Induced Inflammatory Phenotypes in Human Skin

**DOI:** 10.3390/cells12242789

**Published:** 2023-12-07

**Authors:** Kwang-Soo Lee, Yunsik Kim, Jin Hee Lee, Suji Shon, Aram Kim, An Vuong Quynh Pham, Chungho Kim, Dong Hyun Kim, Yoon-Keun Kim, Eun-Gyung Cho

**Affiliations:** 1 H&B Science Center, CHA Meditech Co., Ltd., Seongnam 13488, Republic of Korea; 2 Consumer Health 2 Center, CHA Advanced Research Institute, Bundang CHA Medical Center, Seongnam 13488, Republic of Korea; 3 Department of Dermatology, Bundang CHA Medical Center, School of Medicine, CHA University, Seongnam 13488, Republic of Korea; 4 Department of Life Sciences, Korea University, Seoul 02841, Republic of Korea; 5 MD Healthcare Inc., Seoul 03923, Republic of Korea; 6 Department of Life Science, General Graduate School, CHA University, Pocheon 11160, Republic of Korea

**Keywords:** *Lactobacillus paracasei*, probiotic, extracellular vesicle, tumor necrosis factor-α, anti-inflammation, antiaging, antioxidant, three-dimensional full-thickness skin equivalent

## Abstract

Lactic acid bacteria (LAB), a probiotic, provide various health benefits. We recently isolated a new *Lactobacillus paracasei* strain with strong anti-inflammatory effects under lipopolysaccharide-induced conditions and proposed a new mode of action—augmenting the endoplasmic reticulum stress pathway for anti-inflammatory functions in host cells. The beneficial effects of the *L. paracasei* strains on the skin have been described; however, the effects of *L. paracasei*-derived extracellular vesicles (LpEVs) on the skin are poorly understood. Herein, we investigated whether LpEVs can improve inflammation-mediated skin phenotypes by determining their effects on primary human skin cells and a three-dimensional (3D) full-thickness human skin equivalent under tumor necrosis factor (TNF)-α-challenged inflammatory conditions. LpEVs were efficiently taken up by the human skin cells and were much less cytotoxic to host cells than bacterial lysates. Furthermore, low LpEV concentrations efficiently restored TNF-α-induced cellular phenotypes, resulting in increased cell proliferation and collagen synthesis, but decreased inflammatory factor levels (matrix metalloproteinase 1, interleukin 6, and interleukin 8) in the human dermal fibroblasts, which was comparable to that of retinoic acid, a representative antiaging compound. The beneficial effects of LpEVs were validated in a 3D full-thickness human skin equivalent model. LpEV treatment remarkably restored the TNF-α-induced epidermal malformation, abnormal proliferation of keratinocytes in the basal layer, and reduction in dermal collagen synthesis. Additionally, LpEVs penetrated and reached the deepest dermal layer within 24 h when overlaid on top of a 3D full-thickness human skin equivalent. Furthermore, they possessed superior antioxidant capacity compared with the human cell-derived EVs. Taken together, the anti-inflammatory probiotic LpEVs can be attractive antiaging and antioxidant substances for improving inflammation-induced skin phenotypes and disorders.

## 1. Introduction

The skin has the largest surface area in the human body and is the primary defense tissue and largest immune organ. It protects the body from various harmful environments, such as ultraviolet (UV) radiation, pathogens, and particulate matter, through physicochemical and biological methods. Upon exposure to environmental and physiological stresses, specific epidermal cell types, namely keratinocytes and Langerhans’s cells, trigger primary immune responses by releasing inflammatory cytokines such as tumor necrosis factor (TNF)-α, interleukin (IL)-1, and interferons (IFNs). This evokes downstream cytokines in a cascade from the epidermis to the dermis [1,2,3]. While primary and transient inflammatory responses are crucial for the skin’s early immune reactions, an imbalanced and inadequately resolved inflammatory response can lead to a sustained hyperinflammatory state. Ultimately this imbalance may contribute to various skin disorders, including psoriasis, atopic dermatitis, and skin aging.

The term “inflammaging” refers to chronic, low-grade systemic inflammation that also occurs in the skin and is closely associated with aging, serving as one of the hallmarks of aging [4,5,6,7,8,9]. TNF-α, a key aging-associated proinflammatory cytokine, is upregulated in sustained hyperinflammatory states resulting from imbalanced and unresolved inflammatory responses, thereby contributing to the aging phenomenon [10,11,12]. Conversely, TNFα overexpression is also induced by age-related cellular senescence, indicating the existence of a bidirectional feedback loop between inflammation and aging [13]. In the same context, the long-term TNF-α presence increases reactive oxygen species (ROS) levels and causes premature senescence in the human dermal fibroblasts [6,7], leading to a cycle of harmful effects. Consequently, overexpressed TNF-α reduces dermal fibroblast proliferation, interferes with dermal cell matrix formation by inhibiting collagen synthesis and accelerating collagen and elastin degradation via matrix metalloproteases (MMPs), and upregulates other proinflammatory cytokines such as IL-6 and IL-8, slowing down the healing of damaged skin and aggravating proinflammatory effects, respectively [4,13]. Given its association with skin aging, treating skin cells with TNF-α can be viewed as stimulating inflammation-associated aging processes and thus used to screen and validate the antiaging properties of biomaterials.

Beneficial bacteria such as probiotics provide various health benefits via anti-inflammatory and antimicrobial activity in the human body [14,15]. The skin, the largest organ for microbial cells to colonize, additionally acts as a physical barrier to prevent foreign pathogen invasion while providing a home to the commensal microbiota similar to the gut. Some skin diseases are also associated with an altered microbial state [16,17]. Given that the gut microbiome shapes the host immune system function and exerts systemic metabolic effects [18,19], manipulating the composition and functionality of the intestinal bacterial ecosystem has been suggested to be a potential approach for restoring “altered cellular communication”, another hallmark of aging that results in inflammation [8,9,18]. Therefore, similar to the approach to the gut, the utilization of probiotics or probiotic-derived substances could be an attractive method to improve skin aging by restoring microbial balance and microbial–host cell interactions on the skin and by reducing inflammation levels. In this context, significant attention is currently focused on exploring probiotic-derived extracellular vesicles (EVs) as potent agents for improving cellular communication and modulating inflammatory states [20].

EVs are naturally occurring nanoparticles enclosed by a lipid bilayer and secreted by most living organisms on the earth [21]. Mammalian cell-derived EVs are produced mainly by two different pathways, i.e., direct outward budding of the plasma membrane (ectoderms) and the fusion of multivesicular bodies containing intraluminal vesicles with the plasma membrane and exocytosis (exosome). Essentially, EVs exhibit physiochemical heterogeneity in terms of their origin, size, and molecular composition, while demonstrating functional diversity [22]. Similar to EVs of mammalian cells, most Gram-negative or Gram-positive bacteria release membrane vesicles (henceforth EVs) with sizes ranging from 20 to 400 nm in diameter [23]. Gram-negative bacteria, such as *Escherichia coli*, release various EV types through at least three different routes; however, Gram-positive bacteria, including probiotics, release EVs through one route due to their thick cell walls composed of peptidoglycans [23]. These bacterial EVs affect diverse biological processes of not just bacterial communities but also of host cells and tissues, by mediating the transport of virulence factors, horizontal gene transfer, export of cellular metabolites, and cell–cell communication [20,23,24,25,26]. Particularly, bacterial EVs exhibit immunomodulatory activities and are, therefore, used as vaccines and for developing anticancer drugs [27].

Although bacterial EVs were first discovered in the 1960s in Gram-negative *E. coli* [28], EVs from Gram-positive bacteria have been found recently, and their properties and functions have been actively studied [29]. For example, EVs from Gram-positive bacteria *Staphylococcus aureus* and *Cutibacterium acnes*, which are related to skin inflammatory disorders, atopic dermatitis, and acne vulgaris, respectively, show rapid and strong pathogenic phenotypes compared with their respective parental cells at the same protein concentrations [24,25]. Unlike pathogenic EVs, EVs derived from probiotics such as *Lactobacillus* spp. and *Bifidobacterium* spp. have shown beneficial effects, including the modulation of immune cell differentiation toward an anti-inflammatory phenotype, improvement in inflammatory conditions like atopic dermatitis and food allergies, antidepressant-like effect, and inhibition of cancer cell proliferation [20,30,31,32,33]. One promising probiotic candidate, *Lactobacillus paracasei*, widely present in plants and the stomachs of animals, also increases the production of anti-inflammatory cytokines, decreases the severity of inflammatory bowel disease symptoms, and improves allergies and eczema [34,35,36,37]. Recently, a new *L. paracasei* strain was isolated from the human body (vaginal flora), and its EVs attenuated the intestinal inflammatory response by augmenting the stress pathway of the endoplasmic reticulum [38].

Several studies have investigated *L. paracasei*’s effect on the skin, including scalp and skin diseases. However, studies on the effects of *L. paracasei*-derived EVs (LpEVs) on the skin are lacking. Therefore, we characterized LpEVs derived from *L. paracasei* isolated from the human body and examined their potential to alleviate inflammation-related skin phenotypes by determining their effects in the primary human skin cells. This was also assessed in a three-dimensional (3D) full-thickness human skin equivalent under TNF-α-challenged inflammatory conditions. Thus, we propose that *L. paracasei*-derived EVs can be used as antiaging and antioxidant substances to improve inflammation-dependent aging phenotypes.

## 2. Materials and Methods

### 2.1. Materials

TNF-α, ascorbic acid, and all-trans retinoic acid (RA) (all from Sigma Aldrich, St. Louis, MO, USA) were dissolved according to the manufacturer’s instructions. Cell viability was assessed using EZ-Cytox (DoGenBio Ltd., Seoul, Republic of Korea). The specific protein levels in the culture medium were determined using human enzyme-linked immunosorbent assay (ELISA) DuoSet kits for pro-collagen I alpha 1, total MMP-1, IL-6, and IL-8 (all from R&D Systems Ltd., Minneapolis, MN, USA), according to the manufacturer’s instructions.

### 2.2. Cell Culture

Human dermal fibroblasts (HDFs), human epidermal keratinocytes (HEKs), human adipose-derived mesenchymal stem cells (ADSCs), and human bone marrow-derived mesenchymal stem cells (BM-MSCs) were purchased from ATCC (Manassas, VA, USA). HaCaT cells, a spontaneously immortalized human keratinocyte cell line, were purchased from Thermo Fisher Scientific (Waltham, MA, USA). HDFs and HaCaT cells were cultured in Dulbecco’s modified Eagle’s medium (DMEM; WELGENE Inc., Daegu, Republic of Korea) supplemented with 10% fetal bovine serum (FBS; Thermo Fisher Scientific, MA, USA). HEKs were cultured in EpiLife (Gibco, MA, USA) supplemented with a human keratinocyte growth supplement (HKGS; Gibco, MA, USA). ADSCs and BM-MSCs were cultured in DMEM with low glucose supplemented with 10% FBS. All culture media contained 100 units/mL of penicillin–streptomycin (Thermo Fisher Scientific, Waltham, MA, USA), and all cells were cultured at 37 °C in 5% CO_2_. To prepare EVs derived from ADSCs, BM-MSCs, and HDFs, confluent cells were washed twice with 1× phosphate-buffered saline (PBS) and cultured in serum-free DMEM for 3 d. The conditioned media were collected and passed through a 0.22 μm bottle top filter (Corning, NY, USA) and concentrated approximately 25-fold with a KrosFlo^®^ KR2i TFF System (Repligen, MA, USA) using a 500 kDa KrosFlo hollow fiber filter (Repligen, MA, USA).

### 2.3. Preparation of L. paracasei-Derived EVs

The *L. paracasei* strain used for EV production was previously isolated from the human body, and MD Healthcare Inc. (Seoul, Republic of Korea) reported on its genomic characteristics [38]. LpEVs were prepared as previously described [38]. In brief, *L. paracasei* was inoculated into an EMP medium developed by MD Healthcare and cultured at 37 °C at 200 rpm until the optical density at 600 nm reached 1.0 to 1.5. The culture was subsequently collected and centrifuged at 10,000× *g* at 4 °C. for 20 min to obtain the supernatant. The collected supernatant was passed through a 0.22 μm bottle top filter (Corning, NY, USA) and concentrated 200-fold with a MasterFlex pump system (Cole-Parmer, IL, USA) using a 100 kDa Pellicon 2 Cassette filter membrane (Merck Millipore, Burlington, MA, USA). The concentrates (LpEVs) were again passed through a 0.22 μm bottle top filter and subjected to ultracentrifugation at 150,000× *g* for 3 h at 4 °C. The LpEV protein concentration was measured using a bicinchoninic acid (BCA) protein assay kit (Thermo Fisher Scientific, MA, USA), and the particle size, distribution, and number were analyzed using dynamic light scattering (Zetasizer Nano ZS; Malvern Panalytical, Malvern, UK) and nanoparticle tracking analysis (ZetaView; Particle Metrix GmbH, Ammersee, Germany). The spherical shape of the LpEVs was analyzed using transmission electron microscopy (JEM1011; JEOL, Tokyo, Japan). Bacterial lysates were prepared by adding 1× PBS corresponding to 10% (*w/v*) of the cell weight to *L. paracasei,* which was separated from the supernatant after centrifugation, washed two times with 1× PBS, and sonicated for seven cycles (sonication cycle: Duration_1 min 30 s, Duty cycle (pulse)_30%, Output_5.5). Bacterial whole lysates (LpEX) were centrifuged at 13,000 rpm and 4 °C for 20 min, and the supernatant (LpEX-S) and pellet (LpEX-P) were separated. LpEX-Ps were resuspended in the same PBS volume as the supernatant. The LpEX, LpEX-S, and LpEX-P protein concentrations were measured using a BCA protein assay kit.

### 2.4. LpEV Permeability Assay

LpEVs were incubated with either 2 μg/mL (2.27 μM) DiO (Invitrogen, Waltham, MA, USA) or 6 μM PKH67 (Sigma-Aldrich, St. Louis, MO, USA) dyes for 1 h and 5 min, respectively, at room temperature according to the manufacturer’s instructions. Dye-labeled LpEVs (approximately 10 mL) were placed in a Vivaspin ultrafiltration spin column with a 50 kDa molecular weight cutoff (MWCO) (Merck Millipore, Burlington, MA, USA) and centrifuged at 3600 rpm at 4 °C until the volume reached approximately 0.5–1 mL. To remove the free dye, the concentrated LpEVs were transferred to an Ultra-15 centrifugal filter unit with 10 kDa MWCO (Merck, Darmstadt, Germany), and 10-fold PBS volume was added, followed by centrifugation at 3600 rpm, 4 °C, until a final 0.5–1 mL volume of labeled LpEV was obtained. HaCaT cells were treated with DiO-labeled LpEVs for 4 h, and the culture medium was replaced with a fresh medium. The cells were observed under a fluorescence microscope (IX73 inverted microscope; Olympus, Shinjuku, Japan), and the intensity of fluorescence was determined by reading the signal from the cells using a fluorometer (BioTeK, Winooski, VT, USA). A 3D full-thickness human skin equivalent (see Section 2.7) was treated with PKH67-labeled LpEVs at the center of the epidermal surface for 1 h and washed with PBS. After 6 h or 24 h, human skin equivalents were fixed with 4% formalin, made into a paraffin block, and cut into 5 μm thick slices. Green fluorescence images of the slides were acquired using a Zen 3.1 blue slide scanner (Carl Zeiss, Oberkochen, Germany). The conditioned media of the human skin equivalents at 6 or 24 h were collected and centrifuged at 13,000× *g* rpm at 4 °C for 10 min. Fluorescence intensity was determined using a fluorometer.

### 2.5. Cell Viability Assay

To measure cell viability, HDFs (1 × 10⁴ per well) or HEKs (4 × 10⁴ per well) were cultured in a 48-well plate for 24 h and treated with different concentrations of each sample (LpEV, LpEX, LpEX-P, or LpEX-S) under serum-free conditions. After 24 h, 20 μL of EZ-Cytox was added to each well containing 200 μL of culture media and incubated for 4 h at 37 °C in 5% CO_2_. Absorbance was measured at 450 nm using a microplate reader (INNO-M; LTEK, Seongnam, Republic of Korea). HDFs were cultured for 24 h in a growth medium and additionally incubated under serum-free conditions for 6 h for the cell viability assay after LpEV treatment in inflammatory conditions. TNF-α (20 ng) was first treated for 24 h and each sample at different concentrations was subsequently treated in TNF-α for another 24 h. Culture media were harvested to determine the released protein levels using an ELISA kit. The same volume of fresh medium was added to each well, which was then treated with EZ-Cytox for 4 h, after which the absorbance was measured.

### 2.6. ELISA Assay

The protein levels of pro-collagen I alpha 1, MMP-1, IL-6, IL-8, and IL-1β were analyzed in the culture media of cells treated with TNF-α (20 ng/mL) alone or in combination with RA (2 μM) or LpEV (0.1 μg/mL). Specific ELISA kits for pro-collagen I alpha 1, MMP-1, IL-6, IL-8 (all from R&D Systems, Minneapolis, MN, USA), and IL-1β (Invitrogen, Waltham, MA, USA) were employed for each protein following the manufacturer’s instructions. The detection limits of each ELISA kit were as follows: pro-collagen I alpha 1 (31.2–2000 pg/mL), MMP-1 (62.5–4000 pg/mL, IL-6 (9.38–600 pg/mL), IL-8 (31.2–2000 pg/mL), IL-1β (3.9–250 pg/mL). The conditioned media obtained from the culture of a full-thickness human skin equivalent after sample treatment (as described in Section 2.7) were concentrated to an equal volume for all treatments using a spin column with a 50 kDa MWCO and then subjected to ELISA.

### 2.7. Generation of 3D Full-Thickness Human Skin Equivalent

A reconstituted 3D full-thickness human skin equivalent (human skin equivalent) was generated as previously described [39,40]. Briefly, HDFs were cultured in DMEM supplemented with 10% FBS and 10 mg/mL ascorbic acid for 4 weeks to form a dermal sheet. HEKs were seeded onto a dermal sheet and cultured for one week to build a dermal-epidermal layered structure, which was placed onto another dermal sheet and cultured at the air–liquid interface for 14 d to induce the formation of a full-thickness human skin equivalent. TNF-α (20 ng/mL) was added to the culture medium every other day for 14 d, a period of the air–liquid interface, alone or together with LpEV (1, 10 μg/mL), LpEX (1, 10 μg/mL), or retinoic acid (positive control: 2, 10 μM) to introduce inflammatory skin conditions and examine the recovery effects driven by LpEV. Human skin equivalents were prepared in duplicates for each sample. The culture media of the human skin equivalents were harvested and stored after each treatment to measure the released protein levels using ELISA. After completion of the sample treatment, the human skin equivalents were fixed with 4% formalin (Sigma Aldrich, St. Louis, MO, USA), made into paraffin blocks, and cut into 5 μm thick slices to make slide specimens (the slides), which were subsequently used for staining.

### 2.8. Hematoxylin and Eosin (H&E) Staining

The slides were placed in a staining jar and immersed in absolute xylene (Sigma Aldrich, St. Louis, MO, USA) for 4 min to remove the paraffin. The slides were then sequentially dipped in 100%, 95%, 90%, 80%, and 70% ethanol (Sigma Aldrich), followed by rinsing under running tap water for 2 min. The slides were subsequently stained with Harris hematoxylin (Sigma Aldrich, Taufkirchen, Germany) for 2 min. The slides were then rinsed, treated with 1% hydrochloric acid (Sigma-Aldrich, MO, USA), rinsed again, treated with 1% ammonia, rinsed, and stained with an eosin solution (EMS, Orefield, PA, USA) for 2 min. After rinsing, the slides were sequentially dehydrated in 70%, 80%, 95%, and 100% ethanol, and immersed in xylene for 1 min. Finally, the slides were mounted with Canada balsam (DUKSAN, Seoul, Republic of Korea) and images were acquired using a slide scanner (Axio Scan.Z1; Carl Zeiss, Oberkochen, Germany). The thickness of the epidermal layer in each treatment was measured by dividing the entire image into three consecutive fields, which covered the entire epidermal layer from side to side; a total of six fields per treatment (three fields per treatment, n = 2) were subjected to epidermal thickness measurement. The formula for calculating the thickness (=height) is as follows [41]: *Thickness (Height) of epidermis* = (*Area of epidermal layer*)/(*Width of the region of interest*). Since the width of the region of interest is fixed and kept constant in every condition, the thickness of the epidermis is considered equivalent to the area of the epidermis. The area of interest was marked and automatically measured in ImageJ (https://imagej.nih.gov/ij/index.html, accessed on 18 March 2022). A measurement unit is an arbitrary unit (A.U).

### 2.9. Immunohistochemistry (IHC)

The slides were deparaffinized as described in Section 2.8 to analyze protein expression in human skin equivalents. The slides were washed with a recovery solution of 0.01 M citrate buffer (pH 6.0) and heated three times in a microwave for 5 min. The slides were subsequently kept in PBS for 5 min, incubated in 0.3% H_2_O_2_-methanol solution for 10 min, and rinsed with PBS for 10 min. The slides were treated with a blocking solution (1% bovine serum albumin) for 1 h and incubated overnight at 4 °C with primary antibody (Ki67; Invitrogen, MA, USA). After rinsing with PBS for 10 min, the slides were incubated with the secondary antibody for 1 h, stained with horseradish peroxidase-diaminobenzidine (HRP-DAB; Abcam, Cambridge, UK), and rinsed with PBS for 10 min. The slides were incubated for 1 min, rinsed, and stained with Harris hematoxylin using a DAB substrate kit (Abcam, Cambridge, UK). After rinsing with PBS for 10 min, the slides were sequentially dehydrated in 70%, 80%, 90%, 95%, and 100% ethanol, and immersed in xylene three times for 3 min each. Finally, the slides were mounted using Canada balsam and all images were acquired using a slide scanner.

### 2.10. Masson’s Trichrome Staining

The slides were deparaffinized as described in Section 2.8 to analyze the levels of synthesized collagen in human skin equivalents. Then, the slides were immersed in Bouin’s solution (Sigma-Aldrich, MO, USA) and warmed at 60 °C for 45 min. The slides were washed under running tap water until they turned yellow, and the nuclei were stained with Weigert’s hematoxylin (Abcam, Cambridge, UK) for 8 min, followed by washing. Subsequently, the cytoplasm was stained with an anionic dye (Biebrich scarlet; Abcam), rinsed again, treated with a phosphomolybdic acid (Abcam)-treated mordant solution (Abcam) for 10 min, and immediately stained with an aniline blue solution for 5 min. Finally, the cells were treated with a 1% acetic acid solution for 1 min, rinsed, and dehydrated sequentially in 70%, 80%, 95%, and 100% ethanol. After being immersed in xylene for 1 min, the slides were mounted with Canada balsam, and images were acquired using a slide scanner.

### 2.11. Antioxidants Assay

The antioxidant assay was performed using the oxygen radical absorbance capacity (ORAC) method. In a 75 mM phosphate buffer (pH 7.4), sodium fluorescein (80 nM, 125 μL; Sigma-Aldrich, MA, USA) was mixed with various types of EVs or vitamin C at different concentrations (sample volume 25 μL). Subsequently, 2,2′-Azobis(2-methylpropionamidine) dihydrochloride (AAPH) (75 mM, 50 μL; Sigma-Aldrich) was added, and the mixture was incubated at 37 °C for 60 min. Fluorescence was measured every minute at 485 nm excitation and 528 nm emission. The area under the curve (AUC) was calculated using the formula AUC = 1+ f1/f0 +…+ fi/f0 +…+ f60/f0 (where f0 is the fluorescence value at 0 min and fi is the fluorescence value at i min). The antioxidant capacity for each sample was determined by subtracting the AUC of the negative control from the AUC of that sample (Net AUC). Intracellular ROS levels were determined using 5-(and-6)-chloromethyl-2′,7′-dichlorodihydrofluorescein diacetate, acetyl ester (CM-H2DCFDA), an indicator for ROS in cells. HDFs were treated with CM-H2DCFDA (1 μM), followed by incubation with EVs at different concentrations (0, 1, 10 μg/mL) in the presence of H_2_O_2_ for 1 h. Fluorescence intensity was measured at 485 nm excitation and 530 nm emission using a fluorometer.

### 2.12. Statistical Analyses

Statistical analyses were performed using GraphPad Prism v10.00 (GraphPad, San Diego, CA, USA). For statistical tests, one-way or two-way analysis of variance (ANOVA) with Tukey’s multiple comparison post hoc test was employed unless otherwise stated. Significant differences are represented using asterisks (*, **, ***, or ****) corresponding to the *p* values. Statistical significance was set at *p* < 0.05.

## 3. Results

### 3.1. Characterization and Cell Penetration Activity of LpEVs

**Figure 1:**
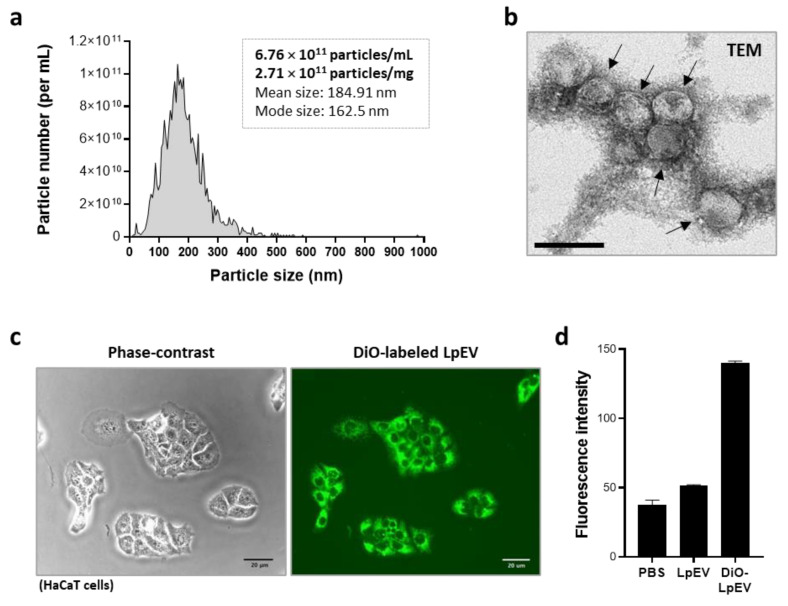
Nanoparticle tracking analysis and cell penetration capacity of LpEVs. (**a**) LpEVs were analyzed using NTA, and the representative analysis data are indicated. (**b**) The representative bio-TEM image of LpEVs (arrows). Scale bar, 100 nm. (**c**) The HaCaT cells were treated with DiO (fluorescent membrane dye; green)-labeled LpEVs for 4 h, after which unabsorbed DiO-labeled LpEVs were washed away. After an additional 3 h incubation, the cell images were taken from live cells under an optical (left) or a fluorescence microscope (right). Scale bar, 20 μm. (**d**) The fluorescence intensity was determined using a fluorometer from the cell culture plate treated with a vehicle (PBS), nonlabeled LpEVs (LpEV), or DiO-labeled LpEVs (DiO-LpEV). LpEVs, *L. paracasei*-derived EVs; NTA, nanoparticle tracking analysis; TEM, transmission electron microscopy; DiO, 3,3’-dioctadecyloxacarbocyanine.

LpEVs attenuate lipopolysaccharide-induced inflammation in the intestine [38]. LpEVs were concentrated using a combination of tangential flow filtration and ultracentrifugation, as previously described [38], to examine their beneficial effects on human skin cells and tissues. Based on the analyses of nanoparticle tracking and bio-TEM, LpEVs had a diameter of <200 nm, with an average value of 190.18 ± 11.13 nm (mode diameter: 149 ± 12.58 nm), implying a wide size distribution although the size of the major population was approximately 150 nm. The mean number of particles per milligram of protein was 2.71 × 10^11^ for purified LpEVs (Figure 1a). According to bio-TEM analysis, LpEVs had a closed spherical membrane structure (Figure 1b), similar to previously described EVs derived from Gram-positive bacteria *L. plantarum* [20,24]. We examined whether LpEVs can be taken up by human skin cells (i.e., the human keratinocyte cell line HaCaT) by labeling LpEVs with DiO, a green fluorescent lipophilic carbocyanine dye. Nonincorporated free dyes were removed during ultrafiltration and diafiltration using a 50 kDa cutoff membrane, by which molecules >50 kDa, including LpEVs, were concentrated, and the labeling buffer was exchanged. We verified that the green signals after treatment came from the DiO-labeled LpEVs, not from free dyes, by performing the same procedure as the DiO-labeled LpEVs for the free dye control. We demonstrated that HaCaT cells treated with DiO filtrates did not display any specific green signals (Figure S1a). In contrast to the DiO filtrates, the DiO and DiO flow-through clearly displayed green signals (Figure S1a). In contrast to the DiO dye control, green signals were only observed in the DiO-LpEV filtrates but not in the nonlabeled LpEV or the DiO-LpEV flow-through (Figures S1a and S2). These data indicated that nonincorporated free dyes passed through the 50 kDa cutoff membrane and the upper DiO or DiO-LpEV filtrates no longer contained free dyes. Therefore, the green signals after treatment with DiO-LpEV filtrates were specific. At 4 h posttreatment with DiO-LpEV filtrates on the HaCaT cells, approximately 100% of the cells demonstrated green fluorescent signals, but not with the buffer control or nonlabeled LpEV (Figure 1c and Figure S2). The fluorescence intensity was measurable, indicating a much higher signal intensity in cells treated with DiO-labeled LpEVs than in cells treated with nonlabeled LpEVs or DiO filtrates (Figure 1d and Figure S1b). These results suggest that the probiotic *L. paracasei*, which lives in the human body, releases EVs that affect human skin cells.

### 3.2. Less Cytotoxic and More Proliferative Effects of LpEVs in the Primary HDFs

**Figure 2:**
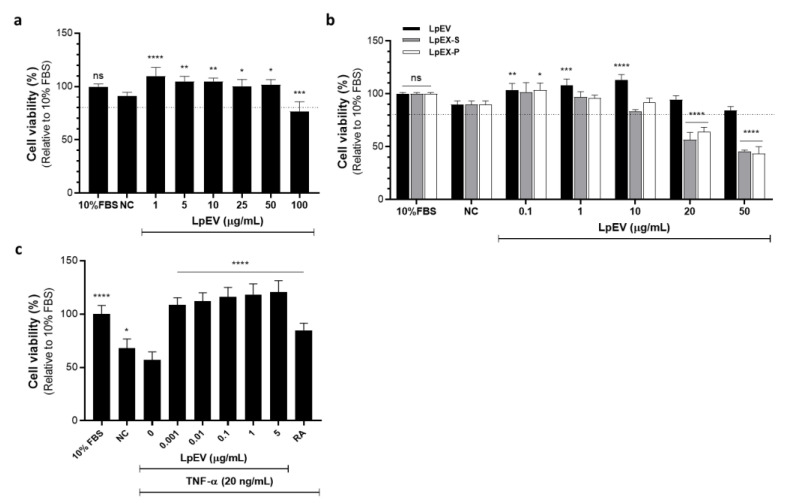
Cell viability after LpEV or LpEX treatment and recovery of cell viability after LpEV treatment in TNF-α-induced inflammatory conditions. (**a**) Human dermal fibroblasts were treated with various LpEV concentrations. (**b**) The supernatant (-S) or pellet (-P) fractions were prepared from sonicated *L. paracasei* lysates (LpEX) via centrifugation. Human dermal fibroblasts were treated with either LpEV, LpEX-S, or LpEV-P at the same protein concentrations. Cell viability was assessed using the water-soluble tetrazolium salt (WST) assay. The dotted lines indicate 80% of cell viability. (**c**) Human dermal fibroblasts were serum starved for 6 h and then treated with 20 ng/mL of TNF-α for 24 h in serum-free conditions. Various LpEV concentrations were treated for an additional 24 h in the presence of TNF-α. Cell viability was determined via the WST assay. As a positive control, 2 μM of all-trans-retinoic acid (RA) was used. The data represent the mean ± standard deviation (n = 6 for (**a**) and (**b**); n = 9 for c; one-way analysis of variance). * *p* < 0.05, ** *p* < 0.01, *** *p* < 0.001, and **** *p* < 0.0001. LpEV, *L. paracasei*-derived EVs; TNF, tumor necrosis factor; NC, serum-free negative control; ns: no significance.

HDFs were treated with various LpEV concentrations under serum-free conditions to investigate whether probiotic LpEVs can stimulate human dermal fibroblasts without inducing cytotoxicity and to seek suitable doses for subsequent experiments. Among the tested concentrations, LpEV treatment did not cause cytotoxicity except at relatively high concentrations (100 μg/mL), which resulted in cell viability of ≤80% (Figure 2a). The recovery of cell viability under serum-free conditions was enhanced at relatively low concentrations (Figure 2a). We compared the effects of LpEVs and bacterial lysates (LpEXs) on cell viability recovery. When the cells were treated with LpEVs, the supernatant (LpEX-S), or the pellet (LpEX-P) of bacterial lysates at the same protein concentrations, LpEVs markedly enhanced cell viability, whereas LpEX-S and -P exhibited cytotoxicity at concentrations at which LpEVs did not induce cellular toxicity (Figure 2b). These results suggest that LpEVs are safer and cause less irritation than bacterial lysates, which are actively used as cosmetic materials.

### 3.3. LpEV Treatment in TNF-α-Induced Inflammatory Conditions Induces Recovery of Cellular Viability

TNF-α is a key inflammatory cytokine in the human skin and mediates an inflammatory reaction cascade in the skin challenged by environmental or internal stressful stimuli. Increased proinflammatory cytokine levels, including TNF-α, IL-1β, and IL-6, are related to skin aging phenotypes [4,6,7]. Therefore, we introduced TNF-α-challenged conditions in the human dermal fibroblasts and examined whether LpEVs can recover TNF-α-induced cellular phenotypes. HDFs treated with TNF-α exhibited a significant decrease in cell viability compared with the nontreated serum-free control (Figure 2c). LpEV treatment at various concentrations significantly restored cellular viability in a dose-dependent manner, exceeding the effect of retinoic acid (RA) at 2 μM (Figure 2c). RA and its precursor retinol (vitamin A) improve aged skin phenotypes such as wrinkles, sagging, and hyperpigmentation [42,43]. Therefore, RA was used as a positive control in our study. The cellular cytotoxicity induced by TNF-α treatment, as well as the recovery effect on cell viability by LpEVs, was evaluated using additional assay systems-lactate dehydrogenase (LDH) release, an indicator of irreversible cell death due to cell membrane damage, and a cell counting kit (CCK)-8, respectively. Cell viability demonstrated a dose-dependent recovery through LpEV treatment in the CCK-8 assay (Figure S3a), and TNF-α-induced cytotoxicity gradually reduced with the increase of LpEV concentrations in LDH release assay, showing an inverse correlation with the increased cell viability (Figure S3b). Based on the result that low LpEV concentrations worked well on human skin cells, we chose an LpEV concentration of 0.1 μg/mL for further study.

### 3.4. LpEV Treatment Leads to the Recovery of Collagen Synthesis but Downregulation of Inflammation-Related Cytokines under TNF-α-Challenged Inflammatory Conditions

**Figure 3:**
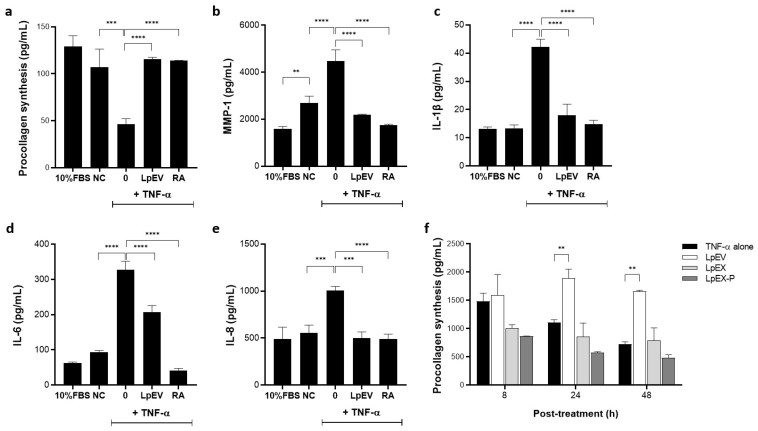
Recovery of TNF-α-inhibited collagen synthesis but downregulation of inflammation-related cytokines by LpEV treatment in human dermal fibroblasts. Human dermal fibroblasts were serum starved for 6 h and subsequently treated with 20 ng/mL of TNF-α for 24 h under serum-free conditions. Cells were treated with LpEV (0.1 μg/mL) or RA (2 μM) for an additional 24 h in the presence of TNF-α. (**a**) The synthesized pro-collagen levels were assessed using a human pro-collagen I alpha 1 ELISA kit. (**b**–**d**) The secreted protein levels of MMP1 (**b**), IL-1β (**c**), IL-6 (**d**), and IL-8 (**e**) were assessed using each specific ELISA kit. (**f**) The conditioned media were harvested at 8, 24, and 48 h posttreatment of LpEV, LpEX, or LpEX-P in the presence of TNF-α. The secreted pro-collagen levels were assessed using a human pro-collagen I alpha 1 ELISA kit. The data are shown as the mean ± SD (n = 3, one-way ANOVA). ** *p* < 0.01, *** *p* < 0.001, **** *p* < 0.0001. NC, serum-free negative control; LpEX, *L. paracasei* bacterial lysates; LpEX-P, LpEX-Pellet; TNF-α, tumor necrosis factor.

When skin cells are exposed to TNF-α, collagen synthesis is inhibited, MMPs are promoted, and other proinflammatory cytokines, such as IL-6 and IL-8, are upregulated [44,45]. We examined whether LpEVs could restore these phenomena induced by TNF-α treatment. Collagen synthesis can be assessed using ELISA, which quantitatively determines the levels of pro-collagen type I N-terminal (PINP) or C-terminal propeptide (PICP) in immature or newly synthesized collagen using specific antibodies [46]. As expected, TNF-α treatment dramatically reduced the new synthesis of collagen; however, LpEV treatment restored collagen synthesis to the level of nontreated control or RA-treated groups (Figure 3a). In contrast to the increase in collagen synthesis, the levels of MMP1 protein, a collagen-degrading enzyme, decreased (Figure 3b). Additionally, the protein levels of senescence-associated inflammatory cytokines IL-1β, IL-6, and IL-8, which are downstream effector molecules of TNF-α and upregulated by TNF-α treatment, were considerably downregulated by LpEVs (Figure 3c–e). Depending on the cytokine, the effects driven by LpEV or RA treatments were different, demonstrating that IL-6 levels were dramatically decreased by RA compared with LpEVs, but those of IL-8 were similarly decreased by LpEV and RA treatments. Given that RA upregulates IL-8 and not IL-6 expression in context- and cell-type-dependent ways [47,48,49], LpEVs showing consistent anti-inflammatory and less irritant effects on skin cells could be good substances for skin care products. Because bacterial EVs are expected to evoke cellular responses in a faster and stronger manner than bacterial lysates [17,18], we compared the effectiveness of LpEVs, LpEX, or LpEX-P at the same protein concentrations by determining the pro-collagen levels over time. At 24 h posttreatment, LpEVs markedly increased collagen synthesis compared with the TNF-α alone-treated group, and this effect was maintained even 48 h after treatment; however, LpEX and LpEX-P failed to induce collagen synthesis within 48 h posttreatment (Figure 3f), suggesting that LpEVs can be a powerful substance compared with its parental cells in terms of evoking beneficial cellular effects such as anti-inflammation and collagen production.

We confirmed the anti-inflammatory effects of LpEVs in human keratinocytes. The primary human keratinocytes and the keratinocyte cell line, HaCaT cells, were cotreated with TNF-α and IFN-γ, which are the first cytokines secreted in an inflammatory response from skin and immune cells and synergistically trigger the downstream inflammatory responses [50,51]. LpEVs at noncytotoxic protein concentrations (approximately 0.1–10 μg/mL) restored cell viability reduced by TNF-α and IFN-γ cotreatment in a dose-dependent manner (Figure S4a–c). Furthermore, the mRNA expression levels of the skin barrier function-related markers, including keratin 14 (*KRT14*), loricrin (*LOR*), involucrin (*IVL*), and aquaporin 3 (*AQP3*), were dramatically downregulated by TNF-α and IFN-γ cotreatment; however, a tendency to recover these phenomena existed after LpEV treatment to a small extent. Interestingly, TNF-α and IFN-γ cotreatment increased the mRNA expression of filaggrin (*FLG*) in the HaCaT cells and the LpEVs reversed this phenomenon (Figure S5). These results suggest that LpEVs have a beneficial effect on the epidermal keratinocytes, which are responsible for the skin barrier function, although LpEV treatment is less effective on epidermal keratinocytes than on dermal fibroblasts.

### 3.5. LpEV Treatment Restores the Epidermal Malformation Induced by TNF-α Treatment in a 3D Full-Thickness Human Skin Equivalent

**Figure 4:**
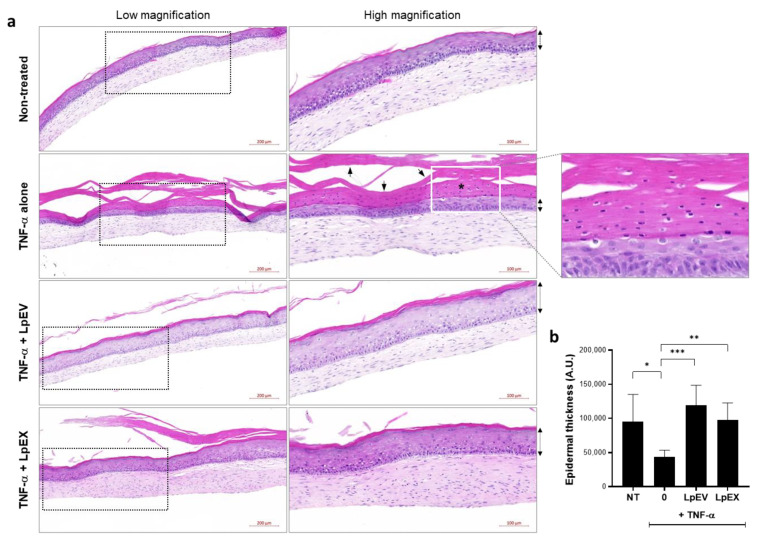
LpEV treatment induces the recovery of TNF-α-induced epidermal malformation in a 3D full-thickness human skin equivalent. (**a**) A 3D full-thickness human skin equivalent was prepared on a 6-week scheme, followed by treatments with retinoic acid (2 μM), LpEV (1 and 10 µg/mL), or LpEX (1 and 10 µg) together with TNF-α (20 ng/mL) 5 times for 2 weeks. The sections (5 μm thickness) of reconstructed human skins were subjected to hematoxylin and eosin stain and scanned using a slide scanner, Zen 3.1 blue. The representative images from 1 μg of LpEV or LpEX-treated reconstructed human skins are depicted. The boxed area in the left panel is enlarged in the right panel. The arrows in the image of the TNF-α alone-treated group (right panel) indicate the malformed epidermal layers, and the asterisk in the white box (enlarged on the right side) represents cells possessing the nuclei in the uppermost layer. Scale bars, 200 μm (left panels) and 100 μm (right panels). (**b**) The thickness of the epidermal layers (indicated by dotted arrows) was determined using Image J. At least two different LpEV batches per two repetitive 3D skin equivalents were analyzed. The data represent the mean ± standard deviation (n = 4; one-way ANOVA). * *p* < 0.05, ** *p* < 0.01, *** *p* < 0.001.

Given that probiotic bacteria-derived EVs modulate the differentiation of host immune cells toward anti-inflammatory phenotypes and improve inflammatory skin conditions [20], we investigated whether LpEVs could improve TNF-α-induced inflammatory phenotypes in a 3D full-thickness human skin equivalent. LpEVs and LpEX were cotreated with TNF-α in human skin equivalents five times for 14 d, a period of the air–liquid interface for epidermal terminal differentiation, and the tissue and culture supernatants were harvested for immunohistological and protein ELISA analyses, respectively. In the TNF-α-treated group, human skin equivalents demonstrated an aberrant epidermal structure characterized by a thin epidermal layer and the presence of nucleated cells in the uppermost layer of the epidermis, which was not typical since these cells were absent in the nontreated control group as expected (Figure 4a, TNF-α alone; arrows in high magnified image). The LpEV treatment completely restored these TNF-α-induced skin phenotypes, demonstrating recovered epidermal thickness and no nucleated cells in the uppermost epidermal layer (Figure 4a; TNF-α + LpEV). Additionally, LpEX demonstrated a recovery effect on the TNF-α-treated human skin equivalent; however, the degree was less than that of LpEV at the same protein concentrations (Figure 4a; TNF-α + LpEX). Epidermal thickness was quantified by measuring the epidermal area (the width per image was fixed and constant; thus, only the area was considered and automatically determined using ImageJ), and the LpEV treatment resulted in the dramatic recovery of the inflammation-induced malformed epidermal structure (Figure 4b).

**Figure 5:**
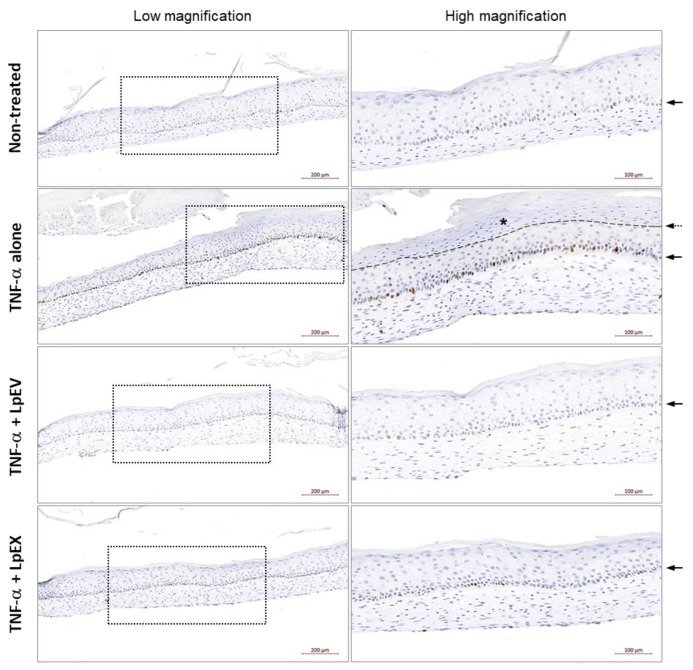
Recovery of TNF-α-induced abnormal cell proliferation by LpEV treatment in the epidermis of a 3D full-thickness human skin equivalent. A 3D full-thickness human skin equivalent was prepared on a 6-week scheme, followed by treatments with retinoic acid (2 μM), LpEV (1 and 10 µg/mL), or LpEX (1 and 10 µg) together with TNF-α (20 ng/mL) 5 times for 2 weeks. The sections (5 μm thickness) of reconstructed human skins were stained with anti-Ki67 antibody and HRP-DAB-conjugated secondary antibody and scanned using a slide scanner, Zen 3.1 blue. The Ki67-positive proliferating epidermal keratinocytes (dark brown color) are shown on the basement membrane (arrows). The representative images from 1 μg of LpEV or LpEX-treated reconstructed human skins are depicted. The boxed area in the left panel is enlarged in the right panel. The upper region of a thin line (asterisk) in the TNF-α alone-treated group indicates a malformed epidermal layer. Scale bars, 200 μm (left panel) and 100 μm (right panel).

Contrary to TNF-α contribution in the phenotypes related to cellular senescence and inflammaging where it reduced the activity of dermal fibroblasts, i.e., proliferation and collagen synthesis [13] as proven in our experiments (Figure 2c and Figure 3), the prolonged TNF-α presence in the epidermis results in the pathogenesis of autoimmune diseases such as psoriasis and psoriatic arthritis by activating keratinocytes and consequently promoting epidermal hyperplasia [52]. Therefore, we examined whether TNF-α treatment induced epidermal proliferation in the basal layer by performing immunostaining with the Ki-67 antibody, which is known to recognize a nuclear antigen present in proliferating cells but is absent in resting cells [53]. Compared with the nontreated control group where the Ki-67-positive proliferating epidermal cells intermittently appeared in the basal layer, the group treated with TNF-α alone showed a clear increase in Ki-67-positive cells (Figure 5; TNF-α alone), ensuring the abnormal epidermal cell proliferation. Notably, the LpEV treatment (TNF-α + LpEV) and, to a lesser extent, the LpEX (TNF-α + LpEX) treatment resulted in the complete disappearance of this phenomenon (Figure 5). Taken together, these results suggest that LpEVs effectively reverse the epidermal phenotypes evoked under the TNF-α-mediated inflammation conditions, which are frequently caused by various environmental cues.

### 3.6. LpEV Treatment Restores the Dermal Collagen Synthesis Reduced by TNF-α Treatment in a 3D Full-Thickness Human Skin Equivalent

**Figure 6:**
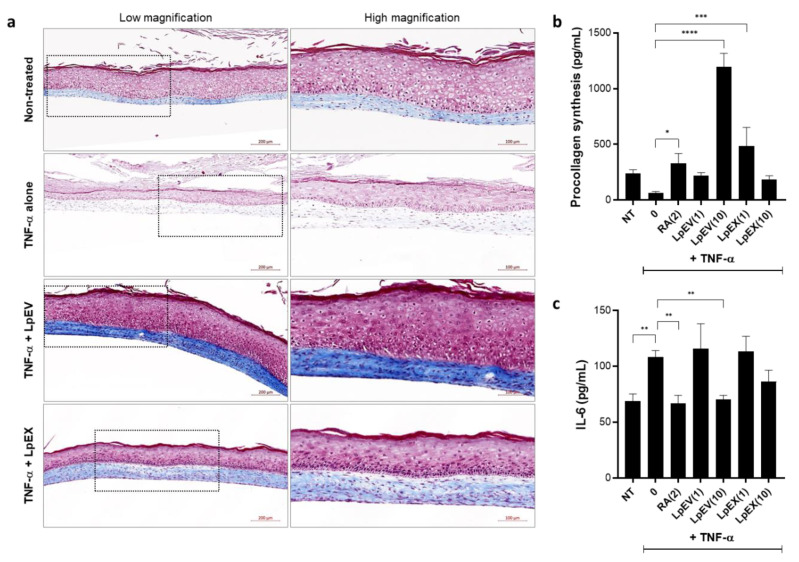
Recovery of TNF-α-inhibited collagen synthesis and inhibition of TNF-α-induced IL-6 secretion by LpEV treatment in a 3D full-thickness human skin equivalent. A 3D full-thickness human skin equivalent was prepared on a 6-week scheme, followed by treatments of retinoic acid (2 μM), LpEV (1 and 10 µg/mL), or LpEX (1 and 10 µg) together with TNF-α (20 ng/mL) 5 times for 2 weeks. (**a**) The sections (5 μm thickness) of reconstructed human skins were stained using Masson’s trichrome (red—keratin; blue—collagen; light red—cytoplasm; black—nuclei) and scanned using a slide scanner, Zen 3.1 blue. The representative images from 1 μg of LpEV or LpEX-treated reconstructed human skins are depicted. The boxed area in the left panel is enlarged in the right panel. Scale bars, 200 μm (left panel) and 100 μm (right panel). (**b**,**c**) The conditioned media were harvested every time the culture medium was changed and subsequently concentrated using Centricons^®^ equipped with a 10 kDa cutoff membrane. The secreted protein levels of collagen or IL-6 were assessed using human pro-collagen I alpha 1 (**b**) and human IL-6 quantikine (**c**) ELISA kits, respectively. The data represent the mean ± standard deviation (n = 3; one-way ANOVA). * *p* < 0.05, ** *p* < 0.01, *** *p* < 0.001, **** *p* < 0.0001.

We further examined whether LpEVs can restore the dermal defects, i.e., collagen reduction, caused by TNF-α treatment following the analyses of recovery effects by LpEV treatment in the epidermis of TNF-α-treated human skin equivalent. Compared with the nontreated control group where collagen signals are shown in light blue, TNF-α treatment caused a drastic reduction in the total collagen levels in the dermis of human skin equivalents (Figure 6a; TNF-α alone). However, LpEV treatment completely restored the reduced collagen levels to a greater extent than nontreated control (TNF-α + LpEV) and to a lesser extent than LpEX (TNF-α + LpEX) (Figure 6a). We assessed the levels of newly synthesized collagen in the conditioned media of human skin equivalents using PINP ELISA. The conditioned medium from each group was collected after each medium change and concentrated to equal volumes. Compared with the nontreated control, TNF-α treatment apparently reduced collagen synthesis; however, the levels between the two groups were not markedly different (Figure 6b). Treatment with RA or low LpEV concentrations restored collagen synthesis to the level of the nontreated control; however, the effect driven by low LpEV concentrations was not significant (one-way ANOVA; *p* = 0.195; Figure 6b). Finally, this effect was evident at high LpEV concentrations, indicating strong collagen synthesis induction (Figure 6b). In contrast to LpEVs, LpEX was less effective at higher concentrations. In addition to measuring newly synthesized collagen levels, we determined the levels of IL-6, a downstream cytokine of TNF-α, using the same concentrated conditioned media. In contrast to the effect on collagen synthesis, IL-6 levels were markedly increased by TNF-α treatment but decreased by RA and high LpEV or LpEX concentrations, although the effect of LpEX on IL-6 levels was insignificant (Figure 6c). These results suggest that similar to the epidermal phenotypes, the dermal phenomena evoked by inflammatory conditions can be effectively restored by LpEV treatment.

### 3.7. Skin Permeability Analysis of LpEVs in a 3D Full-Thickness Human Skin Equivalent

**Figure 7:**
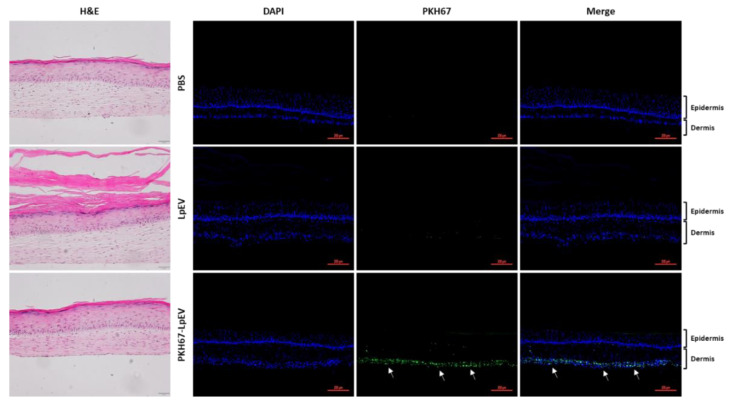
LpEV penetration in a 3D full-thickness human skin equivalent. LpEVs were labeled with PKH67 (green) and ultrafiltrated and diafiltrated using a 50 kDa cutoff membrane. The center of the surface of 3D full-thickness human skin equivalents was overlayed with vehicle (PBS), nonlabeled (LpEV), or PKH67-labeled LpEVs (PKH67-LpEV) for 1 h, followed by washing out with PBS and subsequently incubated for an additional 24 h. Human skin equivalents were fixed and sequential sections from each tissue block were prepared (5 μm thickness). Consecutive sections were stained with hematoxylin and eosin (H&E) or 4′,6-diamidino-2-phenylindole (blue). All images were scanned using a slide scanner, Zen 3.1 blue, and representative images are depicted. Green signals from PKH67 are indicated using arrows. Scale bars, 50 μm (H&E) and 200 μm (immunohistological analysis).

One important role of EVs is to deliver biologically active molecules to nearby or distant cells or tissues; therefore, they are considered natural liposomes or lipid nanoparticles [21]. Based on the characteristics of nanosized vesicles surrounded by a lipid bilayer similar to liposomes, the ability of EVs to penetrate human skin has been a subject of interest. Topically applied MSC exosomes were previously found to be primarily confined to the stratum corneum in normal human skin explants, although the topical application of MSC exosomes to mouse skin alleviated psoriasis-like inflammation [54]. In our earlier study, DiO-labeled LpEVs were easily endocytosed into the human keratinocyte HaCaT cell line within a few hours (Figure 1c,d). Labeled LpEVs were applied onto the top of reconstructed 3D full-thickness human skin equivalents, and fluorescence signals were detected using confocal microscopy to assess the possibility of LpEV penetrating the human skin. LpEVs were labeled with PKH67, a lipophilic membrane dye with green fluorochrome with excitation (490 nm) and emission (504 nm). Nonincorporated free dyes were removed during ultrafiltration and diafiltration using a 50 kDa cutoff membrane, similar to the DiO labeling shown in Figure 1. Nonlabeled or labeled LpEVs were applied to the center of the surface of human skin equivalents for 1 h and subsequently washed with PBS. After 6 or 24 h, the human skin equivalents and culture media were harvested and analyzed using confocal microscopy and fluorometry, respectively. In contrast to the groups treated with either PBS or nonlabeled LpEVs, human skin equivalents overlayed with PHK67-labeled LpEVs demonstrated strong green signals in the deepest dermal layer at 24 h (Figure 7). We further confirmed that after a relatively short incubation time of 6 h, the skin tissue exhibited green signals in both the upper and lower dermal layers (Figure S6a). Finally, fluorescent signals in the culture medium of the PKH67-labeled LpEV group were detected at 24 h but not at 6 h post overlaying (Figure S6b). In summary, these findings suggest that LpEVs can effectively traverse the epidermis and reach the deepest dermal layer within 24 h in reconstructed 3D full-thickness human skin equivalents. This makes them attractive biomaterials with transdermal delivery capabilities when combined with medical devices.

### 3.8. Antioxidant Effect of LpEVs

**Figure 8:**
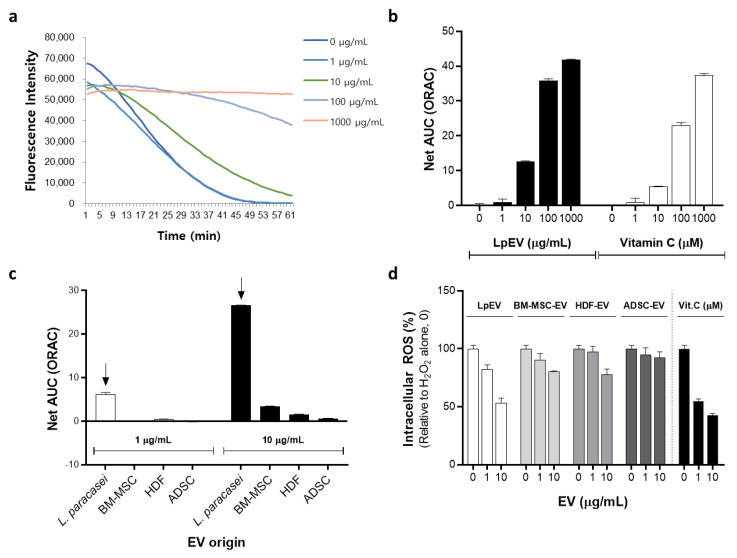
Antioxidant effect of LpEV. The antioxidant activity of LpEV was measured via the oxygen radical absorbance capacity (ORAC) assay. Various LpEV concentrations (0, 1, 10, 100, and 1000 μg/mL) were mixed with a fluorescent probe, which was quenched in the presence of ROS, for 30 min and subsequently incubated with AAPH (ROO• initiator; reactive oxygen species donor) for 60 min. (**a**) Florescent signals were measured every 30 s during the 60 min incubation time. (**b**) The net area under the curve (AUC) was calculated using the following formula: AUCsample − AUCblank. Vitamin C was used as a positive control. (**c**) The antioxidant effects of EVs derived from different origins were compared at the same protein concentrations. LpEVs at low and high concentrations are marked using arrows. (**d**) Intracellular ROS levels were assessed using CM-H2DCFDA, a cellular ROS indicator. HDFs treated with CM-H2DCFDA (1 μM) were incubated with various types of EVs at different concentrations (0, 1, 10 μg/mL) in the presence of H_2_O_2_ for 1 h. Fluorescence signals at 530 nm were measured using a fluorometer. Vitamin C was employed as a positive control The data represent the mean ± standard deviation (n = 4). BM-MSC, Bone marrow-derived mesenchymal stem cell; HDF, human dermal fibroblast; ADSC, adipose-derived stem cell, Vit. C, vitamin C.

Probiotic bacteria, such as *Lactobacillus* spp., have been well reported for their antioxidant potential [55]. We performed an assay of oxygen radical absorbance capacity (ORAC), a method that measures the fluorescent signal from a probe that is quenched in the presence of ROS to determine whether probiotic bacteria-derived EVs (LpEVs) exhibit antioxidant effects. The slope of the fluorescence intensity rapidly decreased with the lowest LpEV concentration and remained almost constant with the highest LpEV concentration (Figure 8a). The antioxidant capacity of LpEVs at each concentration was represented by the net area under the curve (net AUC) (Figure 8b). Additionally, vitamin C, a representative antioxidant, demonstrated a sharp decrease in the net AUC as its concentration decreased. We compared LpEVs with human fibroblast- or MSC-derived EVs using the ORAC assay to determine whether probiotic-derived EVs are more effective than human cell-derived EVs in terms of antioxidant capacity. Interestingly, LpEVs outperformed human cell-derived EVs at all tested concentrations (Figure 8c). Among the human cell-derived EVs, BM-MSC-derived EVs demonstrated a higher antioxidant capacity than ADSC- or HDF-derived EVs. The antioxidant capacity of LpEVs was further evaluated in a cellular system using CM-H2DCFDA dye, a cellular ROS indicator, under H_2_O_2_-treated conditions. As a result, treatment with LpEVs distinctly and dose-dependently reduced intracellular ROS levels, surpassing the antioxidant capacity of human cell-derived EVs. This finding is consistent with the results obtained from the ORAC assay. Thus, LpEVs, which are probiotic EVs, may be potent bioactive substances with anti-inflammatory, antiaging, and superior antioxidant effects compared with human cell-derived EVs.

## 4. Discussion

*Lactobacillus* spp. are representative probiotic bacteria that have been extensively studied in terms of human health and skin care. Among them, *L. paracasei* is beneficial against inflammatory disorders of the intestine and skin; however, studies on the effects of LpEVs on the skin are lacking. Therefore, we verified that LpEVs derived from *L.paracasei* isolated from the human body are potent antiaging substances, exhibiting superior anti-inflammation and antioxidant effects on the human skin cells compared with EVs derived from human mesenchymal stem cells or primary dermal fibroblasts. 

*L. paracasei* used in this study was originally isolated from human vaginal discharge [38]. At birth, the gestational flora of the mother influences the immune development of the early skin and gut flora. Furthermore, the microbiota is initially populated primarily with *Lactobacillus*-dominant flora, which is obtained at birth through the vaginal canal. Interestingly, caesarean-delivered babies harbored bacterial communities similar to those found on the skin surface of the mother and were reported to be more susceptible to allergies and asthma [56,57,58]. Therefore, the probiotic ability of lactobacilli derived from healthy vaginal flora has been of great interest in treating vaginal dysbiosis and for establishing a healthy skin microbiome via immune tolerance in the early immune system. This study suggests that beneficial vaginal bacterial species and their derivatives can be used to fine-tune the imbalanced skin microbiome associated with hyperinflammatory skin disorders.

We used probiotic extracellular vesicles instead of bacterial lysates to investigate the use of probiotic derivatives as potent antiaging substances. Pathogenic bacterial EVs were previously shown to be more effective than bacterial cell lysates in evoking cellular responses in recipient human cells [24,25]. For example, the cellular response, that is, infection-related gene (*IL-6*) expression, in human vessel cells was induced by *S. aureus*-derived EVs (SEVs) within 4 h posttreatment but compared with bacterial lysates at 48 h at the same concentrations, resulting in remarkable differences in the protein levels from 24 h and thereafter. This proves the high efficiency of EVs in delivering the signals to recipient cells [25]. Considering this promising efficiency, we compared probiotic LpEVs and parental cell lysates to determine the cytotoxicity degree and beneficial effects on human skin cells. At relatively high concentrations where LpEV treatment did not exhibit cytotoxicity, treatment with parental cell lysates decreased cell viability (Figure 2b), suggesting that bacterial lysates can cause skin irritation although they are probiotics. We further compared the rate at which each could evoke a beneficial effect, that is, synthesizing collagen. Unlike pathogenic SEVs, the differential effect of LpEVs was observed at 24 h posttreatment; however, that of bacterial lysates was not observed in the 48 h window (Figure 3f). In contrast to the conditions in which pathogenic EVs were directly treated in the cells without additional stimuli, except serum-free conditions, LpEVs and bacterial lysates were treated under inflammatory conditions following serum-free conditions. This suggests that the anti-inflammatory and collagen-promoting effects of LpEVs were driven after inflammation and associated cellular phenotypes had occurred. Given that bacterial EVs, whether pathogenic or beneficial, exhibit stronger and faster effects on recipient cells than bacterial lysates, probiotic EVs could be used as potent, but not irritant, biomaterials in cosmeceutical and medical aesthetics.

Compared with human dermal fibroblasts and the dermis of 3D skin where LpEVs demonstrated strong anti-inflammatory and antiaging phenotypes, the effects of LpEVs were moderate in the human epidermal keratinocytes (HEKS and HaCaT cells) and the epidermis of 3D skin in terms of skin barrier-related phenomena, that is, the expression of skin barrier-related markers (Figures S4 and S5). This implies that preferences exist for EVs toward the target cells. Indeed, EVs that are naturally derived from donor cells possess inherent targeting capabilities that allow them to deliver therapeutic cargo to specific tissues or cells, thereby reducing systemic toxicity [59]. For instance, the tetraspanins on EVs contribute to target cell selection, and neuroblastoma-derived EVs intrinsically express glycosphingolipid glycan groups capable of binding to amyloid-β aggregates in the brain [60,61]. To harness this natural targeting property of EVs, researchers have engineered surface-targeting ligands to enhance specific delivery. This was achieved by inserting the gene encoding the targeting protein into donor cells or by conjugating targeting moieties (RGD derivatives, transferrin) [59,61]. To date, reports claiming that nonanimal cell-derived EVs, that is, plant cell- and bacteria-derived EVs, possess tissue-targeting properties are absent. In addition to the predictable benefits of using nonanimal cells, edible fruits, plants, and probiotic-derived EVs (reliable, scalable, and safe), several trials have been conducted to increase their target effects by loading specific materials [61]. Given that probiotic LpEVs demonstrated differential effects on skin cell types, verifying the targeting molecules responsible for the preferential effects of LpEVs on human dermal fibroblasts instead of keratinocytes would be worthwhile by comparing them with other probiotic EVs demonstrating opposite effects.

Except for omics analyses, most EV studies have been performed using unique EVs from specific cell types; therefore, data comparing the cellular efficacy of EVs derived from different origins are limited. We directly compared the antioxidant capacities of EVs derived from probiotics and mammalian cells, including BM-MSC, ADSC, and HDF. Interestingly, probiotic LpEVs exhibited superior antioxidant capability compared with that of mammalian cell-derived EVs, which was comparable to that of vitamin C (Figure 8). Given that probiotics are considered strong agents in the reinforcement of antioxidant status [62], probiotic-derived EVs may reflect the good properties of parental cells. To date, the factors responsible for the antioxidant properties have not been verified and remain to be determined through proteomic or lipidomic analyses. This finding suggests that probiotic EVs possessing antioxidant, anti-inflammation, and collagen-inducing capacities may be the optimal biomaterials for improving skin conditions under oxidative stress, which is a strong inducer of cell and tissue damage and one of the hallmarks of aging.

When developing skincare products, an important consideration is to increase the transdermal delivery of substances, especially those with large molecular weights and water solubility. Various transdermal delivery techniques have been intensively developed and administered because only a small amount of substances can be delivered through the skin tissue [63]. In addition to active delivery using medical equipment, passive delivery, including microemulsions and vesicles such as liposomes, is frequently utilized to achieve transdermal absorption and sustained release of stored substances. Liposomes are composed of one or more bilayer membranes that separate aqueous media. Their main components are phospholipids with or without cholesterol that mimic cellular membranes [63]. Strikingly different from liposomes, which are synthetic and do not harbor spontaneous targeting ability, EVs are evolutionarily conserved, natural vesicles derived from all living organisms on Earth. This implies that the transdermal delivery and targeting effects of EVs are superior to those of synthetic liposomes. While MSC-derived EVs topically applied are confined primarily to the stratum corneum of the human skin explants [54], our LpEVs quickly penetrated the dermis of the reconstructed 3D full-thickness human skin equivalent (Figure 7). Whether a difference exists in the spontaneous transdermal capacity between human probiotic EVs, in which parental cells inhabit human skin and directly communicate with human skin cells, and human MSC-derived EVs, in which the playground is the dermis, remains to be investigated. EVs applied to the skin surface can easily penetrate the skin layers from the epidermis to the dermis when the surface is damaged due to disorders, diseases, or medical procedures. This characteristic holds promise for utilizing these beneficial EVs as biomaterials for treating and managing various skin phenotypes.

## 5. Conclusions

Herein, we verified that LpEVs derived from the human probiotic *L. paracasei* possess anti-inflammatory and antiaging effects by reversing TNF-α-induced skin phenotypes, including epidermal malformation, dermal collagen reduction, and inflammatory cytokine secretion in vitro and in a 3D full-thickness skin equivalent. LpEVs are superior to human cell-derived EVs in terms of antioxidant activity. Although several EV types derived from different origins exist, data that directly compare the activity and efficacy of differently originated EVs side-by-side are absent. Therefore, LpEVs, which have superior anti-inflammatory and antioxidant activities compared with human adult mesenchymal stem cell- or dermal fibroblast-derived EVs, are promising substances for regenerative medicinal applications to improve skin aging phenotypes and disorders that occur under hyperinflammation and oxidation conditions.

## Data Availability

Data are contained within the article and Supplementary Materials.

## Supplementary Materials

The following supporting information can be downloaded at: https://www.mdpi.com/article/10.3390/cells12242789/s1. Figure S1: Permeability evaluation of LpEVs in the human keratinocyte cell line, HaCaT cells; Figure S2: The cell penetration capacity of LpEVs in the human keratinocyte cell line, HaCaT cells; Figure S3: Recovery of cell viability and cytotoxicity after LpEV treatment in TNF-α-induced inflammatory conditions; Figure S4: Cell viability and recovery effect of LpEV treatment in TNF-α and IFN-γ-cotreated human keratinocytes; Figure S5: Regulatory expression of epidermal differentiation markers by LpEV treatment in TNF-α and IFN-γ-cotreated human keratinocytes; Figure S6: LpEV penetration in a 3D full-thickness human skin equivalent.
