# Supplementary material for: Human Probiotic Lactobacillus paracasei-Derived Extracellular Vesicles Improve Tumor Necrosis Factor-α-Induced Inflammatory Phenotypes in Human Skin
Source: Cells. 2023 Dec 7;12(24):2789. doi: 10.3390/cells12242789 (PMC10741892; doi:10.3390/cells12242789)
Supplement: Supplementary file 1 [file cells-12-02789-s001.zip › cells-2676204-supplementary.pdf]

Supplementary Materials

Human Probiotic *Lactobacillus paracasei*-Derived Extracellular Vesicles Improve Tumor Necrosis Factor-α-Induced Inflammatory Phenotypes in Human Skin

Kwang-Soo Lee, Yunsik Kim, Jin Hee Lee, Suji Shon, Aram Kim, An Vuong Quynh Pham, Chungho Kim, Dong Hyun Kim, Yoon-Keun Kim and Eun-Gyung Cho

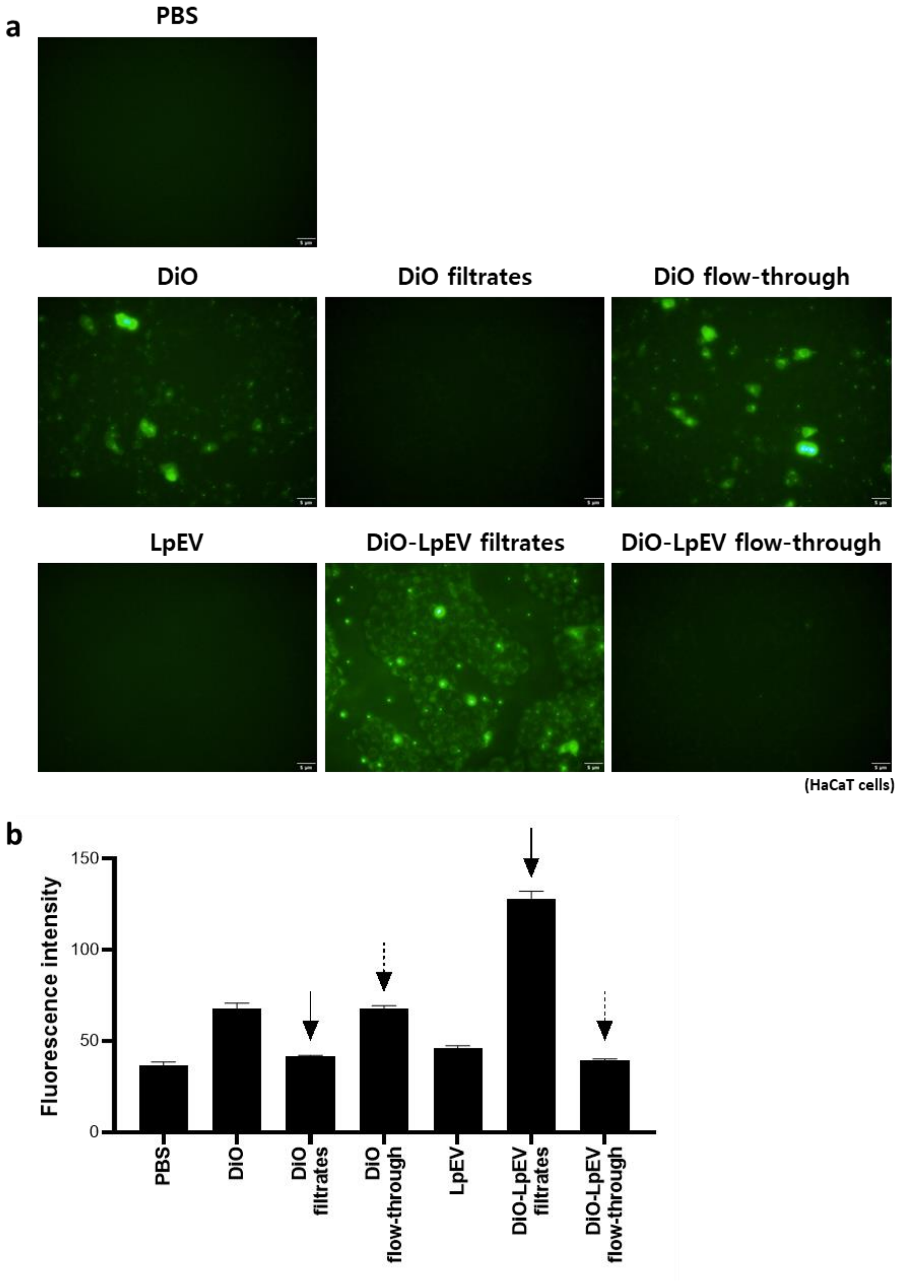

Figure S1: Permeability evaluation of LpEVs in the human keratinocyte cell line, HaCaT cells. (**a**) LpEVs were labeled using a fluorescent membrane dye (DiO). DiO or DiO-labeled LpEVs were concentrated (DiO filtrates or DiO-LpEV filtrates, respectively) via ultrafiltration and diafiltration using a 50 kDa-cutoff membrane. After ultrafiltration, the flow-through fractions of DiO or DiO-LpEV were preserved and used as a control of each process. The HaCaT cells were treated with each fraction for 4 h and unabsorbed DiO or DiO-LpEVs were washed away. After an additional 3 h of incubation, fluorescence images were taken from live cells under a fluorescence microscope. Scale bars, 5 μm. (**b**) The fluorescence intensity was measured using a fluorometer from the cell culture plates treated with each fraction of DiO or DiO-LpEV. DiO, 3,3′-dioctadecyloxacarbocyanine.

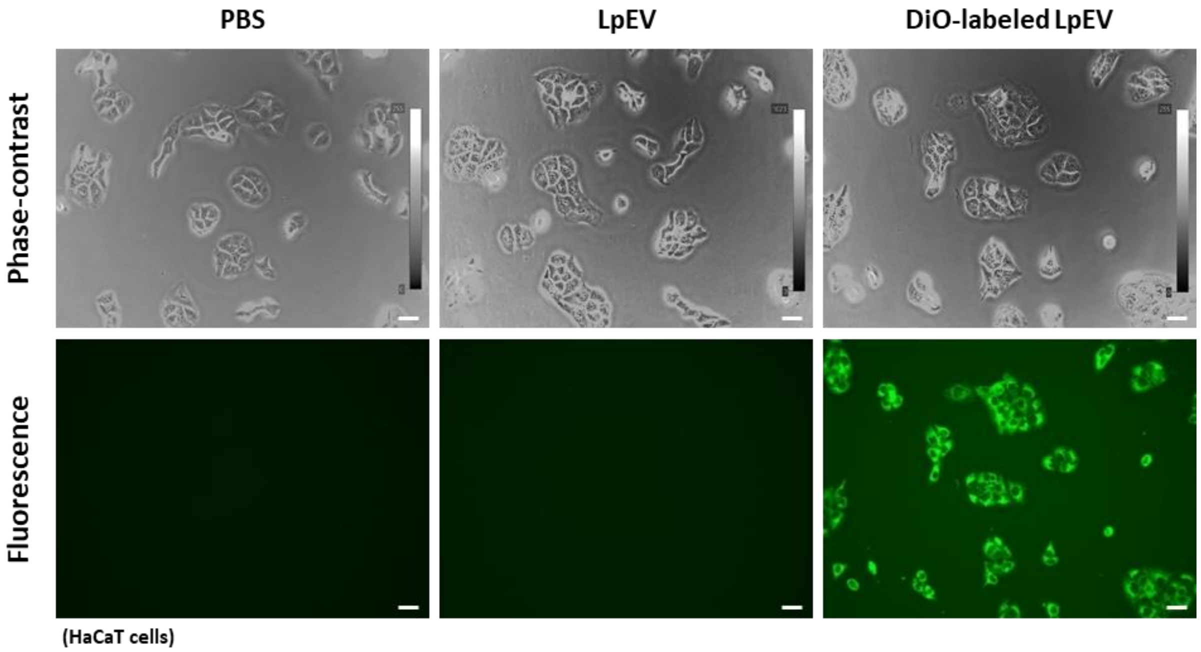

Figure S2: The cell penetration capacity of LpEVs in the human keratinocyte cell line, HaCaT cells. They were treated with a vehicle (PBS), nonlabeled LpEV (LpEV), or DiO-labeled LpEV for 3 h, after which unabsorbed samples were washed away. After an additional 3 h of incubation, the cell images were taken from live cells under an optical (up) or fluorescence microscopes (right). Scale bars, 2 μm.

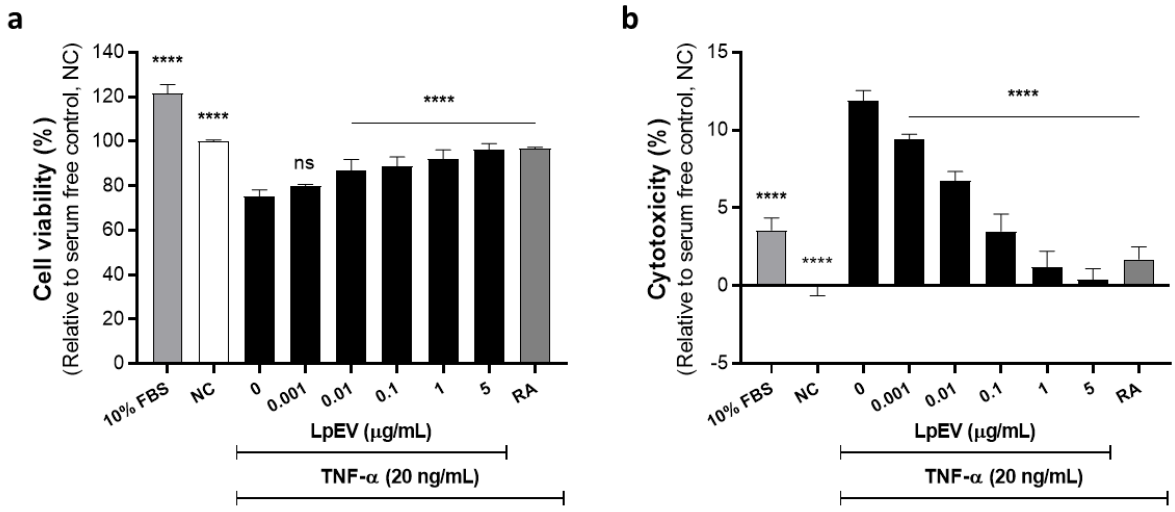

Figure S3: Recovery of cell viability and cytotoxicity after LpEV treatment in TNF-α-induced inflammatory conditions. Human dermal fibroblasts were serum-starved for 6 h and then treated with 20 ng/mL of TNF-α for 24 h in serum-free conditions. Various LpEV concentrations were treated for an additional 24 h in the presence of TNF-α. (**a**) Cell viability was determined via the cell counting kit (CCK)-8 assay. (**b**) Cytotoxicity was determined via the lactate dehydrogenase (LDH) assay from the culture medium. As a positive control, 2 μM of all-trans-retinoic acid (RA) was used. The data represent the mean ± standard deviation (n = 3; one-way analysis of variance). **** $p < 0.0001$. LpEV, *L. paracasei*-derived EVs; TNF, tumor necrosis factor; NC, serum-free negative control; ns, not significant.

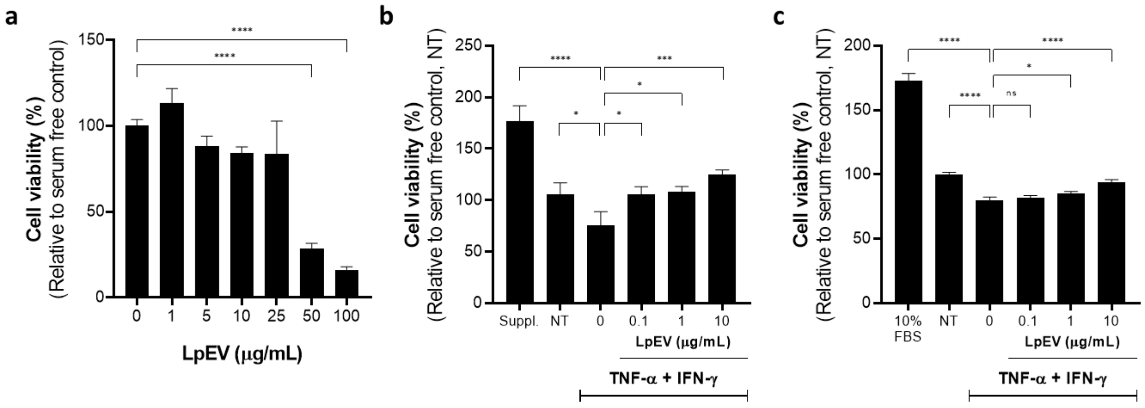

Figure S4: Cell viability and recovery effect of LpEV treatment in TNF-α and IFN-γ-cotreated human keratinocytes. (**a**) Primary human epidermal keratinocytes (HEKs) were supplement-starved for 24 h and subsequently treated with various LpEV concentrations for another 24 h. Cell viability was assessed using the water-soluble tetrazolium salt (WST) assay. (**b**) HEKs were starved for 6 h and then cotreated with TNF-α (40 ng/mL) and IFN-γ (20 ng/mL) for 24 h under supplement-free conditions. Cells were treated with different LpEV concentrations (0.1, 1, and 10 μg/mL) for another 24 h in the presence of TNF-α and IFN-γ. (**c**) HaCaT cells were serum-starved and subjected to the same experimental treatments as that of HEKs in (**b**). The effect of cellular recovery effect by LpEV treatment was determined through the WST assay. The data represent the mean ± standard deviation (n = 3; one-way ANOVA). * $p < 0.05$, *** $p < 0.001$, **** $p < 0.0001$.

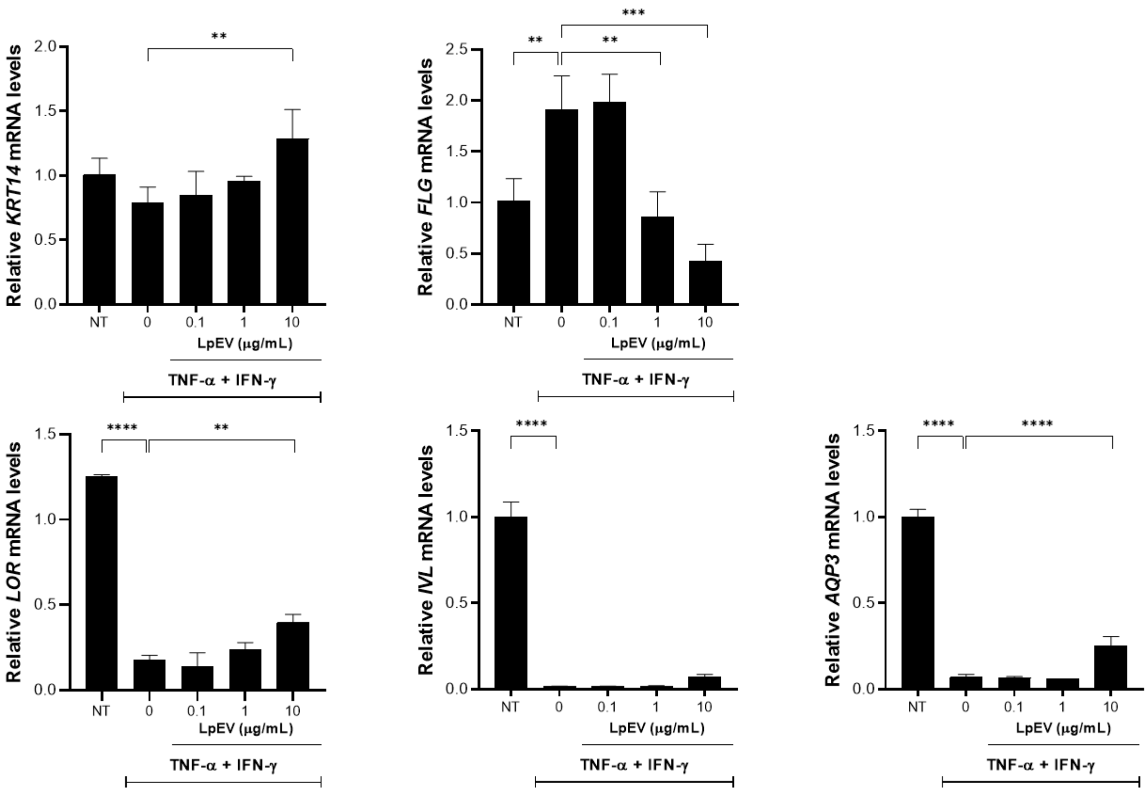

Figure S5: Regulatory expression of epidermal differentiation markers by LpEV treatment in TNF-α and IFN-γ-cotreated human keratinocytes. HaCaT cells were serum-starved for 6 h and subsequently treated with TNF-α (40 ng/mL) and IFN-γ (20 ng/mL) for 24 h under serum-free conditions. Cells were treated with different LpEV concentrations (0.1, 1, and 10 μg/mL) for another 24 h in the presence of TNF-α and IFN-γ. Cells were directly harvested with Trizol solution after washing with PBS and the total RNAs were prepared and used for cDNA synthesis via reverse transcription. The mRNA expression level of each gene (*KRT14*, *FLG*, *LOR*, *IVL*, and *AQP3*) was assessed via real-time polymerase chain reaction using a gene-specific TaqMan probe. Each gene expression was normalized by GAPDH levels. The relative expression levels were calculated using the formula, ΔΔCt. The data represent the mean ± standard deviation (n = 3; one-way ANOVA). ** $p < 0.01$, *** $p < 0.001$, **** $p < 0.0001$.

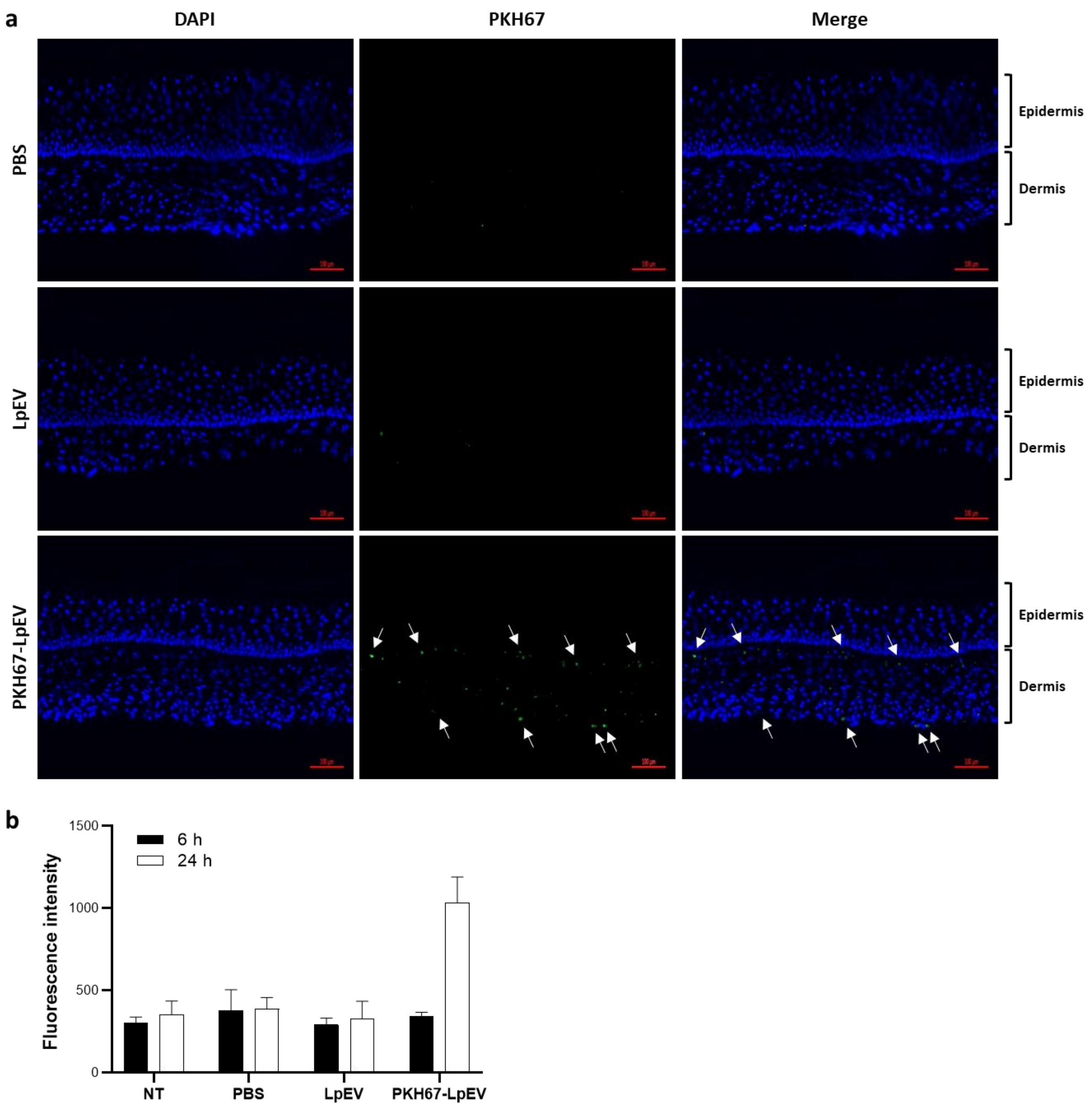

Figure S6: LpEV penetration in a 3D full-thickness human skin equivalent. LpEVs were labeled with PKH67 (green) and ultrafiltrated and diafiltrated using a 50 kDa-cutoff membrane. (**a**) The center of the surface of 3D full-thickness human skin equivalents was overlayed with a vehicle (PBS), nonlabeled (LpEV) or PKH67-labeled LpEVs (PKH67-LpEV) for 1 h, followed by washing out with PBS, and subsequently incubating for additional 6 h. Human skin equivalents were fixed and sequential sections from each tissue block were prepared (5-μm thickness). Consecutive sections were stained with 4′,6-diamidino-2-phenylindole (blue). All images were scanned using a slide scanner, Zen 3.1 blue and representative images are depicted. Green signals from PKH67 are indicated using arrows. Scale bars, 100 μm. (**b**) The conditioned media of human skin equivalents were collected at 6 or 24 h after treatments in different groups and the fluorescence intensity was measured using a fluorometer.
